# Factors Associated with Quality of Life among Hemodialysis Patients in Malaysia

**DOI:** 10.1371/journal.pone.0084152

**Published:** 2013-12-16

**Authors:** Nor Baizura Md. Yusop, Chan Yoke Mun, Zalilah Mohd Shariff, Choo Beng Huat

**Affiliations:** 1 Department of Nutrition and Dietetics, Faculty of Medicine and Health Sciences, Universiti Putra Malaysia, Serdang, Selangor, Malaysia; 2 Department of Medicine, Manipal Medical Collage, Melaka, Malaysia; University of Florida, United States of America

## Abstract

Although hemodialysis treatment has greatly increased the life expectancy of end stage renal disease patients, low quality of life among hemodialysis patients is frequently reported. This cross-sectional study aimed to determine the relationship between medical history, hemodialysis treatment and nutritional status with the mental and physical components of quality of life in hemodialysis patients. Respondents (n=90) were recruited from Hospital Kuala Lumpur and dialysis centres of the National Kidney Foundation of Malaysia. Data obtained included socio-demography, medical history, hemodialysis treatment and nutritional status. Mental and physical quality of life were measured using the Mental Composite Summary (MCS) and Physical Composite Summary (PCS) of the Short-Form Health Survey 36-items, a generic core of the Kidney Disease Quality of Life Short Form. Two summary measures and total SF-36 was scored as 0–100, with a higher score indicating better quality of life. Approximately 26 (30%) of respondents achieved the body mass index (24 kg/m^2^) and more than 80% (n=77) achieved serum albumin level (>35.0 mg/dL) recommended for hemodialysis patients. The majority of respondents did not meet the energy (n=72, 80%) and protein (n=68,75%) recommendations. The total score of SF-36 was 54.1±19.2, while the score for the mental and physical components were 45.0±8.6 and 39.6±8.6, respectively. Factors associated with a higher MCS score were absence of diabetes mellitus (p=0.000) and lower serum calcium (p=0.004), while higher blood flow (p=0.000), higher serum creatinine (p=0.000) and lower protein intake (p=0.006) were associated with a higher PCS score. To improve the overall quality of life of hemodialysis patients, a multidisciplinary intervention that includes medical, dietetic and psychosocial strategies that address factors associated with mental and physical quality of life are warranted to reduce further health complications and to improve quality of life.

## Introduction

End stage renal disease (ESRD) is a recognised public health problem worldwide [1]. The increasing prevalence of ESRD parallels the increasing prevalence of type 2 diabetes mellitus. The total number of people with diabetes is projected to rise from 336 million in 2012 to 522 million in 2030 [2]. In South Asia, particularly India and Pakistan, diabetic nephropathy is the second highest cause of ESRD [3,4]. There is increasing evidence that diabetes mellitus (DM) patients have higher incidence rate of dialysis compared to their non-DM counterparts [5,6]. It is thus projected that the incidence of ESRD will increase in the future due to the “diabetic epidemic” [7].

The most preferred treatment modality for ESRD in Malaysia is hemodialysis (HD). The prevalence of ESRD patients on dialysis has tripled from 7837 in 2001 to almost 23,000 in 2010 [8]. Hemodialysis improves serum creatinine, albumin and prealbumin, normalises the protein catabolic rate (nPCR) as well as increases the dietary intake of patients [9,10]. Despite its advantages, HD is highly associated with malnutrition and lower quality of life (QOL) [11,12]. Severe malnutrition among HD patients is reported to be approximately 4.6% - 19%, while 72% - 90.9% are mildly malnourished [11-14].

Quality of life (QOL) in patients with ESRD is influenced by the types of renal replacement therapy. Patients who underwent kidney transplant (KT) achieved better QOL compared to dialysis patients [15]. Wu et al in a follow-up (prospective) study found that HD patients showed greater improvement in SF-36 domain scores than peritoneal dialysis patients [16]. Among HD patients, the mental health and physical health dimensions of QOL are strongly associated with morbidity and mortality [17,18]. Nutritional status is an important factor that determines the QOL of patients undergoing dialysis treatment. Body mass index (BMI), cholesterol, serum albumin, hemoglobin and dietary intakes may influence QOL [19,20]. Other factors associated with QOL of HD patients are HD duration, age, ethnicity [21-23].

In Malaysia, information on QOL among HD patients is limited. Most studies conducted were on QOL and its associated factors in different health conditions such as asthmatic, coronary artery bypass and thalassaemia patients [24-26]. A limited number of studies have examined the relationship between socio-demographics, treatment characteristics and nutritional status with QOL among hemodialysis patients [12,27]. However, these studies did not assess comprehensive factors associated with QOL. Thus, the present study aimed to determine the relationship between medical history, hemodialysis treatment and nutritional status with quality of life in hemodialysis patients. We hypothesized that the QOL of HD patients could be influenced by the presence of co-morbidities and current treatment and nutritional status. 

## Methods

### Respondents

This cross-sectional study was carried out at selected dialysis units in Hospital Kuala Lumpur (HKL) and the National Kidney Foundation of Malaysia (NKF) in Klang Valley. The location of the study was determined after taking into account several factors such as location of the dialysis centres and accessibility to HD patients. Selangor and the Federal Territory have the highest number of dialysis centres with 96 out of 347 or 27.7% of the HD centres in Malaysia. A total of 165 respondents meeting the inclusion criteria (aged 18 years and above, had received HD for at least three months and were in stable condition) were recruited into the study. A stable condition was defined as no hospital admission due to renal complication at least three months prior to data collection. 

Out of 97 respondents who agreed to participate, 90 had complete data. Data for five respondents were not complete; two respondents (2.1%) withdrew from study. Ethical approval and permission to conduct the study were obtained from the Medical Research Ethics Committee of Universiti Putra Malaysia and the Medical Research Ethics Committee of HKL and NKF, respectively. Written informed consent was obtained from the respondents prior to data collection. A pre-announcement of the study was performed by the staff nurse in charge prior to data collection. Chinese enumerators were recruited to assist in the data collection. They administered the questionnaires to respondents who were unable to complete the assessment (questionnaire and diet recall) due to physical impairment, such as poor vision or limited manual dexterity, or difficulty in understanding the Malay language. 

### Measurements

#### Anthropometrics

Weight, height, mid-arm circumference (MAC), mid-arm muscle circumference (MAMC), skin fold and calf circumference measurements were performed by trained investigators and using standardised techniques [28]. These assessments were conducted within 10-20 minutes after the HD session and was taken on the side of the body which did not have a vascular access or a cast. All anthropometric measurements were taken twice, and the average was calculated. Respondents were weighed using a TANITA HD-309 digital scale before and after the HD session of a midweek dialysis session. Weight was measured with the respondents wearing normal light, indoor clothing and without shoes to obtain the most accurate reading. The reading was recorded to the nearest 0.1 kg. Height was obtained using SECA Body Meter 208 and recorded to the nearest 0.1 cm. During the height measurement, respondents were without shoes and were required to stand erect with their feet together and eyes in a parallax state. The measurement of knee height was used for elderly respondents who have difficulties standing or maintaining an erect posture as knee height is not affected by height loss due to vertebral compression. Height and weight measurements were used to calculate body mass index (BMI). 

Mid-arm circumference at a non-vascular access site and calf circumference were obtained using a fibreglass tape. Both measurements were recorded to the nearest 0.1 cm. Mid-arm circumference was used to calculate MAMC [29]. The skinfold measurements at four sites (triceps, biceps, subscapular and iliac crest) were determined using a Lange skinfold calliper (Cambridge Instrument, Cambridge, MA, USA) and recorded to the nearest 1 mm. 

Respondent was asked to stand with his/her feet together for all skinfold measurements. In order to measure skinfold thickness for triceps, the upper most edge of the posterior border of the acromion process of the scapular was marked using a cosmetic pencil. Then, the tape measure was held and extended down the posterior surface of the arm to the tip of the olecranon process. The mid point (at the back) between the acromion and the olecranon process was marked. A fold of skin and subcutaneous adipose tissue was grasped gently with the thumb and forefingers, approximately 1.0 cm above the point at which the skin was marked, with the skin-fold parallel to the long axis of the upper arm. Bicep was measured same as for the triceps, but with the measurement of the biceps skinfold at the front of the upper arm (instead of the back, as with the triceps). The level was the same as for the triceps and circumference, and the location was in the midline of the anterior part or the arm.

The measurement method of subscapular skinfold was the same as for the biceps and triceps. Then, the inferior angle of the right scapula was palpated. A fold of skin and subcutaneous adipose tissue was grasped directly below (1.0 cm) and medial to the inferior angle. Skinfold measurement at suprailiac required respondent to abduct arms slightly to improve access to the side. The measurement was taken at the iliac crest. The skin was grasped at an oblique angle, just posterior to the midaxillary line below the natural cleavage lines of the skin [29]. 

Body density was calculated using the formula of Durnin and Womersley, while the percentage of body fat was calculated using Brozek’s equation [30,31]. The measurement values were then compared to the recommendations of Medical Nutrition Therapy for Chronic Kidney Disease (Table 1) [32]. 

**Table 1:** Recommended anthropometry and dietary intake for patients on hemodialysis.

Measurement	Recommendation
Anthropometry	
BMI (kgm-2)	> 24
Triceps (mm)	>50th percentile
Mid-arm circumference (mm)	>50th percentile
IDWG (kg)	2.0 – 3.0 kg
Dietary Intake	
Energy	35 kcal/kg body weight if <60 years of age
	30-35 kcal/kg body weight if >60 years of age
Protein	1.2 g/kg BW
Carbohydrate	50-60% of energy intake
Fat	25-35% of energy intake
Sodium	2 – 3 g
Potassium	2 – 3 g
Phosphate	800 – 1000 mg
Fluid	750 to 1000 ml/day

Medical Nutrition Therapy for Chronic Kidney Disease (2005)

#### Subjective Global Assessment

The Subjective Global Assessment (SGA) has been used in dietetics practices to assess nutritional status [33]. Subjective global assessment focuses on specific features of the subject’s history and physical examination. Specific features under history include history of weight loss during the previous six months (described as both kilograms and proportionate weight loss), dietary intake in relation to the usual pattern (classified as normal or abnormal intake), presence of significant gastrointestinal symptoms (anorexia, nausea, vomiting, and diarrhoea), functional capacity (bedridden to full capacity) and the metabolic demands of the patient's underlying disease state.

Loss of subcutaneous fat (as measured in the triceps region and the mid-axillary line at the level of the lower ribs) and muscle wasting in the quadriceps and deltoids (determined by loss of bulk and tone that is detectable by palpation) were determined through the physical examination. These two physical features were rated as either normal (0), mild (1+), moderate (2+), or severe (3+). The subjects were then classified as (A) well-nourished, (B) moderate or suspected malnutrition and (C) severe malnutrition. The SGA was performed immediately after the HD treatment, taking the hydration status into account. 

#### Dietary Intake

Dietary intake was assessed using a 24-hour diet recall (non-dialysis day) and one day food record (dialysis day). Respondents were required to recall and record all food and beverages consumed. They were also requested to eat as usual and not to change their usual intake. A food album and a set of household measurements (glass, soup bowl, plate, cup, teaspoon and tablespoon) were used to guide respondents in estimating portion sizes [34]. Complete instructions on how to estimate and record portion sizes of foods and beverages were also provided to the respondents. For those who could not read or understand the instructions, assistance was sought from family members. Phone call interviews for diet recall were also conducted upon request. Both the recall and record forms required respondents to provide information on meal time, food items and ingredients, estimated amount, brand name and method of food preparation. Dietary data were then analysed using Nutritionist Pro (First Data Bank, USA Inc.) software. The adequacy of energy and nutrient intakes was determined using the recommendations of Medical Nutrition Therapy for Chronic Kidney Disease as shown in Table 1 [32].

#### Quality of Life (QOL)

Quality of life was measured using Short-Form Health Survey 36-items (SF-36), a generic core of Kidney Disease Quality of Life Short Form (KDQOL-SF^TM^) [35]. The SF-36 instrument consists of 36 items. However, only 35 items, representing eight scales and two summary measures, were utilised in this study. The two summary measures are the physical component summary (PCS) and mental component summary (MCS). The physical component summary comprises scales of pain (2 items), physical functioning (10 items), general health perception (5 items) and role limitations caused by physical health problems (4 items). The four scales of MCS were role limitations caused by emotional health problems (3 items), social functioning (2 items), emotional well-being (5 items) and energy/ fatigue (4 items) [35]. 

The scoring for SF-36 was based on the KDQOL-SF™ Version 1.3 Scoring Program (v 3.0), which yields a score for each scale, two summary measures and total SF-36. Each scale is scored as 0-100, with a higher score indicating better QOL. The scores of the two summary measures and the total SF-36 are based on the average of the respective scale components. The Cronbach’s alpha coefficient for the 35 items was 0.923. 

#### Other variables

Secondary data on demographic factors, medical information and HD treatment were obtained from the respondent’s medical report and dialysis record. The required socioeconomic and demographic information were age, gender, ethnicity (Chinese, Malays and other), education level (no formal education, primary school, secondary school and tertiary education), current employment status (unemployed, government, private and part time) and current estimated household income (monthly). 

 There were four items that were related to the respondent’s health status - primary renal diagnosis, co-morbidities, prescribed medications and prescribed vitamins or minerals. For the type of co-morbidities disease, cardiovascular disease (CVD) was defined as documentation of any of the following conditions in the respondents’ medical record: coronary heart disease/coronary artery disease, peripheral vascular disease, acute myocardial infraction and congestive heart failure. The medical information was obtained directly from the respondent’s medical report and uncertainties were clarified with the staff nurse in charge. Information on HD techniques was obtained from the respondent’s medical report and dialysis book. HD techniques was defined as information related to the HD procedure and treatment. The clinical information included duration of dialysis, rate of blood flow, urea kinetics (Kt/V) and nPCR.

Biochemical data obtained from the medical report comprised of renal function tests (serum calcium, serum phosphate and serum creatinine), liver function tests (serum albumin), electrolytes (serum potassium and serum sodium) and lipid profile (total cholesterol, high-density lipoprotein (HDL), low-density lipoprotein (LDL) and triglycerides (TG)). The latest measurement prior to the data collection was used and compared with the normal range [32]. Uncertainties were clarified with the staff nurse in charge as well as with the respondents. 

### Data Analysis

Statistical analyses were performed using SPSS software version 19.0. Descriptive statistics including means, ranges, standard deviation and frequencies were used to present the respondent’s demography, clinical characteristics, SGA, HD technique, QOL scores, energy and nutrient intakes. Anthropometry data and biochemical data were compared with the recommended value from the Medical Nutrition Therapy for chronic kidney disease (2005) [32]. Multivariate linear regression was conducted to identify factors associated with physical and mental components of QOL among HD patients. Logarithmic conversion was performed for data not normally distributed. In all steps of the analysis, p<0.05 was considered significant.

## Results

The respondents comprised 44 (48.9%) males and 46 (51.1%) females with a mean age of 49.7 ± 14.1 years (Table 2). Chinese (n = 62, 68.9%) were the largest group recruited, followed by Malays (n = 19, 21.1%) and others (n = 9, 10.0%). As the majority of the respondents were unemployed (n = 64, 71.1%), only a small group had a household monthly income of more than RM 3000 (n = 21, 23.3%). The most frequent aetiologies of ESRD were unknown causes (n = 30, 33.3%), followed by DM (n = 20, 22.2%), glomerulonephritis (n = 7, 7.8%), hypertensive nephrosclerosis (n = 7, 7.8%) and systemic lupus erythaematosus (n = 6, 6.7%). There was no further documentation on the type of diseases or conditions that contributed to the unknown causes in the respondents’ medical records. The exact diagnosis is not known. All respondents were dialysed three times/week for four hours per session, even those with a monthly income of RM 2000 or less. Since 2001, the Malaysian government has been subsidising RM 50 for each HD treatment for low-income patients receiving HD treatment at government and non-government dialysis centres [36]. The average duration of dialysis of the respondents was 55 ± 39 months, ranging from 4 – 56 months. The mean Kt/V was 1.6 ± 0.5, and average blood flow was 297.1 ± 38.5 ml/minute.

**Table 2:** Background characteristics of hemodialysis respondents (n=90).

Characteristics	n (%)	Mean ± S.D
Socio-demography		
Age (years)		49.7 ± 14.1
24 – 34	14 (15.6)	
35 – 49	30 (33.3)	
50 – 64	28 (31.1)	
≥ 65	18 (20.0)	
Gender		
Males	44 (48.9)	
Females	46 (51.1)	
Ethnicity		
Chinese	62 (68.9)	
Malay	19 (21.1)	
Others	9 (10.0)	
Educational Level		
No formal education	10 (11.1)	
Primary school	33 (36.7)	
Secondary School	40 (44.4)	
Tertiary education	7 (7.8)	
Current employment status		
Unemployed	64 (71.1)	
Government	2 (2.2)	
Private	14 (15.6)	
Part time	10 (11.1)	
Current estimated household income (monthly)		
<RM 1000	22 (24.5)	
RM 1000 - RM 2000	28 (31.1)	
RM 2001 - RM 3000	19 (21.1)	
> RM 3000	21 (23.3)	
Medical History		
Primary renal diagnosis		
Unknown cause*	30 (33.3)	
Diabetes mellitus	20 (22.2)	
Glomerulonephritis	7 (7.8)	
Hypertensive nephrosclerosis	7 (7.8)	
Systemic Lupus Erythaematosus (SLE)	6 (6.7)	
Others**	10 (11.1)	
Presence of co-morbidities		
Hypertension	62 (68.9)	
Diabetes mellitus	23 (25.6)	
Cardiovascular disease	7 (7.8)	
Number of prescribed medications		
Vitamin and mineral supplements	85 (94.4)	
Calcium carbonate	79 (87.8)	
Erythropoietin (EPO)	45 (50.0)	
Hypercholesterolaemia drugs	41 (45.6)	
Antihypertensive drug	35 (38.9)	
Oral diabetic agent (ODA)	18 (20.0)	
Insulin	5 (5.6)	
HD treatment		
Duration of dialysis (months)	90	55.2 ± 39.0
Blood flow (ml/minute)	90	297 ± 39
Kt/V	90	1.6 ± 0.5
nPCR (g/kg/day)	56	1.1 ± 0.3

^*^ Exact diagnosis is not known.

^**^ Other causes include obstructive nephropathy and toxicity.

The mean dry weight and body mass index (BMI) of respondents were 57.7 ± 13.0 kg and 22.8 ± 4.7 kgm^-2^, respectively (Table 3). Approximately 26 (30%) of the respondents achieved the recommended BMI for HD patients, which is BMI ≥ 24 kg/m^2^ [32]. The mean for triceps skinfold (TSF) was 13.3 ± 6.2 mm, and mid-arm circumference (MAC) was 27.1 ± 4.4 mm. Approximately 72 (80%) and 31 (34%) of the respondents had TSF and MAC less than the 50^th^ percentile [32]. Only one respondent was severely malnourished. The respondent was a female who complained of being physically weak and was experiencing loss of appetite. More than half (n = 49, 54.4%) of the respondents were mildly-moderately malnourished while the others (n = 40, 44.4%) were well-nourished.

**Table 3:** Nutritional status of hemodialysis respondents.

Measurement	Mean ± S.D.	n (%)	Recommendation*	n (%)	
				Less than recommendation	More than recommendation
Anthropometric					
Dry weight (kg)	57.7 ± 13.0				
BMI (kgm^-2^)	22.8 ± 4.7		> 24.0	64 (71.1)	26 (28.9)
Triceps (mm)	13.3 ± 6.2		>50th percentile	72 (80.0)	18 (20.0)
Biceps (mm)	7.7 ± 4.7				
Subscapular (mm)	14.3 ± 6.1				
Suprailiac (mm)	14.1 ± 6.3				
MAC (mm)	27.1 ± 4.4		>50th percentile	31 (34.4)	59 (65.6)
MAMC (mm^2^)	23.1 ± 3.6				
Calf circumference (cm)	33.2 ± 3.6				
Percentage of body fat (%)	26.4 ± 7.1				
Subjective Global Assessment			Well nourished	50(55.5)	40 (44.4)
Well nourished		40 (44.4)			
Mild-moderately malnourished		49 (54.4)			
Severely malnourished		1 (1.1)			
Biochemical data					
Serum calcium (mmol/L)	2.3 ± 0.2		2.0-2.6	87 (96.7)	3 (3.3)
Serum phosphate (mmol/L)	1.8 ± 0.6		0.8-1.6	39 (43.3)	51 (58.6)
Serum creatinine (mg/dL)	3.7 ± 4.8				
Serum albumin (mg/dL)	39.9 ± 4.3		>35	13 (14.4)	77 (85.5)
Serum potassium (mmol/L)	4.9 ± 0.8		3.5 - 5.5	70 (77.8)	20 (22.5)
Serum sodium (mmol/L)	139.0 ± 3.0				
Total cholesterol (mmol/L)	4.9 ± 1.1		<5.2	64 (71.1)	26 (28.8)
HDL (mmol/L)	1.1 ± 0.3		>1.2	65 (72.2)	25 (27.8)
LDL (mmol/L)	2.8 ± 0.7		<3.8	82 (91.1)	8 (8.9)
Triglycerides (mmol/L)	2.1 ± 1.3		<1.6	47 (52.2)	43 (47.8)
Dietary intake					
Energy (kcal/kg BW)	26.8 ± 7.0				
age < 60 years	26.9 ± 6.1		≥ 35	54 (60.0)	13 (14.4)
age ≥ 60 years	24.3 ± 7.0		30 - 35	18 (20.0)	5 (5.6)
Protein (g/kg BW)	1.1 ± 0.4		1.2	68 (75.6)	22 (24.4)
Carbohydrate (g)	213.4 ± 51.6		50-60% of energy intake	55 (61.1)	35 (38.9)
Fat (g)	42.8 ± 14.2		25-35% of energy intake	87 (96.7)	3 (3.3)
Sodium (mg)	1943 ± 986		2000-3000 mg/day	78 (86.7)	12 (13.3)
Potassium (mg)	909 ± 303		2000-3000 mg/day	90 (100.0)	
Phosphate (mg)	736 ± 312		800-1000 mg/day	74 (82.2)	16 (17.8)
Calcium (mg)	250 ± 116		<1500 mg/day	90 (100.0)	
Fluid intake (ml)	1112 ± 214		750 - 1000 ml/day	28 (31.1)	62 (68.9)

* Medical Nutrition Therapy Guidelines for Chronic Kidney Disease (2005)

The mean serum calcium and serum phosphate were 2.3 ± 0.2 mmol/L and 1.8 ± 0.6 mmol/L, respectively. The majority of respondents (n = 87, 96.7%) had a normal serum calcium level. More than half of the respondents (n = 51, 58.6%) had elevated serum phosphate. Approximately 13 (14.4%) of respondents had a serum albumin level lower than 35.0 mg/dL. The mean total cholesterol, high-density lipoprotein (HDL), low-density lipoprotein (LDL) and triglycerides (TG) were 4.9 ± 1.1 mmol/L, 1.1 ± 0.3 mmol/L, 2.8 ± 0.7 mmol/L and 2.1 ± 1.3 mmol/L, respectively. There were 26 (28.8%) respondents with elevated total cholesterol and 43 (47.8%) respondents with elevated TG. Most respondents (n = 65, 72.2%) had HDL below the recommended range (> 1.2 mmol/L), but LDL (n = 82, 91.1%) in the recommended range. 

Based on the MNT, the recommendation for energy intake for respondents more than 60 years old is 30-35 kcal/kg body weight and for those who are less than 60 years old is 35 kcal/kg body weight [32]. In the study, the mean total energy intake was 26.8 ± 7.0 kcal/kg body weight, with a higher mean intake in respondents aged less than 60 years old (26.9 ± 6.1 kcal/kg body weight) than for 60 years and above (24.3 ± 7.0 kcal/kg body weight). Approximately 72 (80%) of respondents had inadequate energy intake. For protein, the mean intake was 1.1 ± 0.4 g/kg, with 68 (75.6%) of the respondents failing to achieve the recommended protein intake (1.2 g/kg) [32].

The overall scores of the SF-36 are presented in Table 4. The mean total score for SF-36 was 54.1 ±19.2, while the mean for PCS and MCS were 39.6 ± 8.6 and 45.0 ± 8.6, respectively. For PCS, pain had the highest score (66.9 ± 24.3), while role limitations-physical had the lowest score (30.3 ± 38.5). For the MCS, the highest score was social function (66.8 ± 26.0) and the lowest score was role limitations - emotional (49.3 ± 45.9). It shows that the kidney disease had a greater effect on physical health than on mental health. 

**Table 4:** Quality of Life (QOL) of hemodialysis respondents (n=90).

SF – 36	Score* Mean ± S.D.
Total score of SF-36	54.1 ±19.2
Physical Component Summary (PCS)	39.6 ± 8.6
Pain	66.9 ± 24.3
Physical functioning	61.6 ± 22.0
General health	43.3 ± 18.4
Role limitations—physical	30.3 ± 38.5
Mental Component summary (MCS)	45.0 ± 8.6
Social function	66.8 ± 26.0
Emotional well-being	63.7 ± 17.3
Energy/fatigue	50.8 ± 18.2
Role limitations—emotional	49.3 ± 45.9

^*^ For each scale, summary measure and total score, the score ranges from 0 - 100, with a higher score indicating better QOL.

Table 5 shows the relationship between medical history, HD treatment and nutritional status factors with the QOL of HD patients. Factors associated with a higher MCS score were absence of diabetes mellitus (p=0.004) and lower serum calcium (p=0.016), while higher blood flow (p=0.000), higher serum creatinine (p=0.000) and lower protein intake (p=0.006) were associated with a higher PCS score. 

**Table 5:** Factors related to QOL in hemodialysis respondents.

QOL summary measures	B (Unstandardised Coefficients)	Std. Error	Beta (Standardised Coefficients)	p-value
MCS**^*a*^**				
DM	-6.383	2.149	-.324	0.004
Serum calcium	-11.088	4.489	-.270	0.016
PCS**^*b*^**				
Log of blood flow	20.828	5.554	.338	0.000
Log of serum creatinine	2.826	.636	.401	0.000
Protein intake	-8.009	2.833	-.255	0.006

^a^ R = 0.377, Adj. R^2^ = 0.120; F= 6.226, P = 0.003.

^b^ R = 0.641, Adj. R^2^ = 0.387; F= 17.219, P = 0.000.

PCS = physical component summary and MCS = mental component summary

a and b model adjusted for age, gender, race and medication

## Discussion

There was comparable disposition among males and females in this study. In the study, DM (n = 20, 22.2%) and unknown causes (n= 30, 33.3%) were the two main primary renal diagnoses among the respondents. A study conducted at the east coast of Malaysia found that 48% of diabetic patients had proteinuria and 16% microalbumia, which are the earliest indicators of diabetic kidney disease [37]. In this study, the mean Kt/V was 1.6 ± 0.5, which was higher than the recommended value, and the majority of the respondents (88.8%) were adequately dialysed. Tarek et al found that achieving a Kt/V of 1.5 was a more suitable target for HD patients because it might be an avenue for improving the neuromuscular functions of patients [38]. 

In the study, the increase in the percentage of HD patients with higher BMI (more than 25 kg/m^2^) was due to the increased number of HD patients with diabetes as a consequence of diabetic nephropathy [27]. On the other hand, Ohkawa et al reported that the composition of muscle mass, fat mass and fat distribution differed among HD patients with an increased number of years on HD treatment [39]. Ishimura et al stated that although HD patients may have satisfactory BMI, their ratio of muscle mass and fat mass changed as an outcome of HD treatment [40]. Higher BMI, hypercholesterolaemia, hypercreatininaemia and hyperhomocysteinaemia were reported as protective factors against cardiovascular disease among HD patients in contrast to the general healthy population [41]. According to the Subjective Global Assessment (SGA), the majority of the subjects were mildly malnourished (54.4%) or well nourished (44.4%). Only one subject was severely malnourished. The results of this study were comparable to those of Jones et al. [14]. A total of 69% of their patients were well nourished, 30.5% mildly nourished and none was severely malnourished. In contrast, Rutledge and McMahon documented a high percentage of malnourished patients in their study (19% severe, 25% moderate and 28% mild malnutrition) [11]. 

Elevated serum phosphate level indicates hyperphosphataemia. Elevation of serum phosphate was mainly caused by dietary non-compliance [42]. Consistent hyperphosphataemia was associated with the development of myocardial hypertrophy and poor bone health [43]. Only a small percentage of the respondents had elevated serum calcium. Overall, the mean serum calcium and serum phosphate were comparable to the 13^th^ Report of the Malaysian Dialysis and Transplant Registry at 2.3 mmol/L and 1.8 mmol/L, respectively [27].

In this study, the majority of respondents had low HDL levels, and about half of the respondents had elevated TG levels. The results were consistent with the study by Piperi et al. [44]. Increased cholesterol level among HD patients has been strongly associated with the development of CVD, which is one of the causes of death among HD patients [45]. The low mean HDL values may also be due to lack of physical activity, as most of the HD patients were inactive due to the effects of the HD treatment [46]. 

The majority of the respondents failed to achieve the recommendation for both energy and protein intake. This result was consistent with Bossola et al [47]. They reported that the percentage of patients who did not achieve the recommended intake of energy was higher than the percentage of the patients who did not achieve the intake of protein. They also found a small portion of the patients did not achieve the recommended intake for both energy and protein. Decreased energy intake was negatively associated with the presence of anorexia and increase of age in HD patients [47]. 

Daily mean intake of phosphate was high, while none of the respondents had excessive intake of potassium. Durose et al found that patients had difficulties in reducing the intake of food high in phosphate [42]. However, they had complied with reduced intake of food high in potassium. As a consequence, serum phosphate was elevated but serum potassium remained in the normal range. The intake of fluid was higher in 68.9% of the respondents. This was comparable with the results of Kugler et al., who reported that the degree of non-adherence of fluid intake was high (74.6%) among their respondents [48]. 

Patients on HD treatment are at risk of having poor quality of life [12]. Previous studies have shown that the mental and physical health dimensions of quality of life displayed a strong association with morbidity and mortality [15-18]. In the present study, physical and mental quality of life among HD patients is influenced by several health and nutritional factors.

Tanaka et al showed that HD patients with high serum calcium (>2.6 mmol/L) were more likely to have poor mental health. They found that a significantly lower mental health score was identified in patients with corrected calcium > or = 11 mg/dl than in <8.4 (P = 0.04), > or =8.4 to <10.2 (P = 0.009) and > or =10.2 to <11 mg/dl (P = 0.003). The association was significant even after adjustment for age, sex and other confounders [49]. However, the mechanism involved in the association between serum calcium and the mental dimension of quality of life has not been adequately addressed in clinical studies. Several studies have reported that elevated serum calcium was associated with increased mortality in dialysis patients because it increased the risk of cardiovascular diseases [50,51]. Kalantar-Zadeh et al showed that mortality was strongly associated with a low mental dimension of QOL among HD patients [17]. Therefore, for patients on HD, serum calcium in the low-normal range is important to minimise the complications associated with high serum calcium and to improve quality of life [49-51]. 

The present study found that serum calcium was negatively associated with MCS of QOL. There are limited studies on the effects of low serum calcium on mental health status or mortality [17,50,52]. According to Miller et al, high (> 2.5 mmol/L) calcium levels was consistently associated with mortality. Whereas, association between low (<2.25 mmol/L) calcium and mortality was more prominent among patients with higher serum phosphorus (1.1 mmol/L) and PTH (> 150 pg/ml) [52]. In our study, 64.4% of respondents had serum calcium in the range of 2.25 - 2.5 mmol/L. As the majority of respondents had serum calcium within the normal range, this could explain the better MCS of QOL with lower serum calcium observed in this study.

The present study showed that the absence of DM is associated with better mental quality of life. Previous studies reported that non-diabetic patients had better MCS compared to DM patients [53,54]. It was found that a combination of DM and chronic medical condition (renal failure) might adversely affect the mental dimension as measured by SF-36 [54]. This association could be explained by the better overall health status of HD patients without DM compared to those with DM. Diabetic patients on HD have a higher burden of morbidity and mortality due to risk of microvascular and macrovascular such as cardiovascular diseases, cerebrovascular events and peripheral vascular disease than non-diabetic patients on dialysis [55]. Several studies found that a combination of DM and chronic medical conditions such as renal failure might adversely affect mental health status such as vitality, social functioning and role-emotional as measured by SF-36 [56,57]. 

We found that higher blood flow and serum creatinine as well as lower protein intake were associated with better physical quality of life. In the present study, 63.3% of the respondents had blood flow of 300 ml/minute and above. High blood flow is correlated with Kt/V, which is a measure of dialysis adequacy [57]. The variations in blood flow and dialysate flow were positively related to the variation of the indexes Kt/V and protein catabolic rate (PCR) and consequently to the HD adequacy [56]. Defining a Kt/V of 1.2 as a minimum level of dialysis adequacy (K/DOQI, 2000), 88.9% of the respondents were adequately dialysed. Borzou et al showed that increasing the blood flow rate by 25% is effective in increasing Kt/V [57]. Patients with average Kt/V levels of greater than or equal to 1.3 had better HRQOL as measured by signiﬁcantly higher scores (p<0.05) in 6 of 8 SF-36 domains (physical functioning, general health perceptions, emotional well-being, role-emotional, social function, and energy/fatigue) [58].

Similar to the results of Kalantar-Zadeh et al, the present study showed that a high serum creatinine level is associated with better PCS of QOL [59]. The serum creatinine level is proportionate to dietary protein intake and somatic (skeletal muscle) mass. As only a small percentage of HD patients in this study (10 %) were not adequately dialysed (10%) and had adequate protein intake (24%), serum creatinine should be more indicative of somatic protein concentration (muscle mass) rather than high protein intake [60]. Allen et al also found that an increase in serum creatinine was associated with the increase in the PCS of quality of life and Karnofsky Index (KI) scores. In their study, physical functioning tasks listed in the PCS and assessed by KI were focused on the physical functioning of the lower extremity that is related to mobility [60]. It was concluded that the association between high serum creatinine and better PCS was due to the better status of muscle composition, especially in the lower limbs. In a study among pre-transplant patients, Streja et al documented that larger pre-transplant muscle mass, reflected by higher pre-transplant serum creatinine level, was associated with better graft and patient survival [61].

Protein is an important nutrient to be considered in the management of HD patients. HD patients are required to follow the recommended protein intake of 1.2 g/kg body weight to attain better nutritional status [28-32]. Ohri-Vachaspati and Sehgal showed that inadequate protein status or protein intake, reflected by low serum albumin level and low protein catabolic rate, was independently associated with poor QOL [62]. In another study, Morsch et al found that low serum albumin was associated with low physical functioning [63]. Kalantar-Zadeh et al also showed that patients with a higher serum albumin level had better quality of life in terms of physical and mental health [17]. 

In the present study, we found that low protein intake was associated with better PCS. Further analysis of patients with adequate and inadequate protein intake revealed that the inadequate protein group had better mean serum albumin, better BMI status (not significantly different) and high PCS score (t=-2.857, p=0.004) compared to the adequate protein intake group. Although the inadequate protein group did not achieve protein intake recommendation, they still managed to have good nutritional status, which could contribute to better PCS.

Another explanation for the low protein intake and better PCS observation in this study is related to the accuracy of reporting of protein intake by the HD patients given that the use of diet recall and records is not without limitations [60]. It has been suggested that Normalised Protein Catabolic Rate (nPCR) is a better method for protein intake assessment as it is a valid and clinically useful measure of protein degradation and protein intake in dialysis patients [26]. Kloppenburg et al suggested that an estimation of dietary protein intake and dietary energy intake required at least three PCR measurements or seven and five food recording days, respectively [64]. 

Several points should be considered in interpreting the results. First, is the accuracy of the 24-hours dietary intake and food record, which is highly dependent on respondent’s memory and cooperation from the family members to complete the food record. A recommended method to assess the validity of dietary intake, especially dietary protein intake is the Protein Equivalent of Total Nitrogen Appearance (PNA) or Protein Catabolic Rate (PCR). Protein Catabolic Rate (PCR) is a valid and clinically useful measure of net protein degradation and protein intake in the maintenance of dialysis patients [28]. Kloppenburg et al suggested that an estimation of DPI and DEI required at least three PCR measurements or seven and five food recording days respectively [64]. Therefore, in future, it is important to include measurement of PCR.

Second, to assess the effect of under-reporting of dietary intake in this study, basal metabolic rate of respondents was calculated using the predictive equation by the World Health Organization (WHO) (FAO/WHO/UNU, 1985) [65]. The ratio of energy intake (EI) to BMR was calculated. The ratio of EI to BMR of less than 1.2 is not compatible with habitual intake and normally signifies under-reporting of energy intake [66]. From the study, the mean for the ration EI to BMR was 1.13 ± 0.24. Only 38% of the respondents had EI:BMR exceeding 1.2. Hence, the majority of the respondents were under-reporting their dietary intake. 

In future, a longitudinal study is required in order to observe the determinants of poor QOL and associated factors. In addition, a comphrehensive assessment consists of Protein Equivalent of Total Nitrogen Appearance (PNA) or Protein Catabolic Rate (PCR), repeated biochemical analysis and details analysis on body composition such as Dual Energy X-ray Absorptiometry (DEXA) should be included. There is growing evidence of a potential link between hypercalcemia and mental health among HD patients. It is recommended that future studies should delineate the pathophysiology or mechanism relating hypercalcemia to mental health as well as long-term implications of hypercalcemia on HD patients. Despite these limitations, our findings that poor quality of life among HD patients is associated with co-morbidities, poor nutritional status and inadequate treatment, are consistent with those reported in other settings [17,18]. These findings confirm the universal importance of addressing these factors in efforts to improve health and quality of life of HD patients. 

In conclusion, the present study provides an understanding of factors that are associated with QOL in HD patients. It is important for health professionals to emphasise that low blood flow, DM, increased serum calcium and low level of serum creatinine factors can potentially impair the overall QOL of the HD patients. Low QOL among HD patients is closely associated with higher risk of morbidity and mortality . With the rising prevalence of DM and ESRD worldwide, there could be increasing demand for diabetes-related ESRD treatment, particularly hemodialysis [3-8]. The development and implementation of multidisciplinary interventions consisting of psychosocial and specific medical and dietetic strategies that focus on factors associated with mental and physical quality of life are warranted to prevent further health complications and to improve quality of life of hemodialysis patients. 
